# Association between RS3763040 polymorphism of the AQP4 and idiopathic intracranial hypertension in a Spanish Caucasian population

**DOI:** 10.1515/tnsci-2022-0309

**Published:** 2023-09-11

**Authors:** Juan José Tellería-Orriols, Samsara López-Hernández, Inmaculada Vidriales-Vicente, Carlos Alberto Rodríguez-Arias

**Affiliations:** Unit of Excellence Institute of Biology and Molecular Genetics (IBGM), University of Valladolid and Spanish National Research Council (CSIC), Valladolid, Spain; Medina del Campo Hospital, Medina del Campo, Spain; Clinic Biochemistry, University Clinic Hospital of Valladolid, Valladolid, Spain; University Clinic Hospital of Valladolid, Valladolid, Spain

**Keywords:** idiopathic intracranial hypertension, aquaporin-4, SNP, single-nucleotide polymorphisms

## Abstract

**Background:**

Idiopathic intracranial hypertension (IIH) is a condition of increased intracranial pressure of unknown aetiology. Principal symptoms are headache, visual disturbances, and obesity, together with elevated intracranial pressure. Unspecified MRI, despite normal ventricle size, suggests alterations in the water flux cellular mediated by the brain water channel aquaporin-4 (AQP4). The association among IIH, cerebral spinal fluid malfunction, reabsorption, and functional or regulatory modifications of AQP4 is a hypothesis not confirmed.

**Methods:**

Blood samples were collected from 72 Spanish Caucasian patients with IIH. A genetic association study was performed with bi-allelic SNPs rs1049305 and rs10244884 in AQ1 and rs2075575, rs3763043, and rs3763040 in AQ4. Genetic data were compared with 94 healthy Caucasian control. Statistics studies were assessed by Pearson’s *χ* ^2^ tests for 2 × 2 (alleles) or 3 × 2 (genotypes) contingency tables. A *P* value < 0.05 was considered to be statistically significant.

**Results:**

Statistically significant differences were found when comparing the results of the rs3763040 polymorphism of the AQ4 locus of IIH patients with controls, in genotypic frequencies (*P* = 0.0442) and allele frequencies (*P* = 0.0171). Furthermore, a statistically significant difference (*P* = 0.0207) was found in individuals carrying and not carrying the minor allele (GG + GA individuals vs GG homozygotes). No statistically significant differences were found when comparing allele and genotypic frequencies for SNPs rs1049305 and rs10244884 of AQ1 and rs2075575 and rs3763043 of AQ4.

**Conclusions:**

The association of AQP4 and specifically of its polymorphic variant rs3763040 with IIH should be validated in other ethnic groups in order to assess more precisely the role of AQP4 in the etiopathogenesis of IIH.

## 1 Introduction

Idiopathic intracranial hypertension (IIH), also known as pseudotumour cerebri, is a rare neurological disorder characterized by increased intracranial pressure (ICP) over 25 cm/H_2_O or 18 mm/Hg, in the absence of hydrocephalus or intracranial pathology, and otherwise normal cerebrospinal fluid (CSF) contents [1,2].

The etiology of IIH remains unknown. Although this disorder was once called benign intracranial hypertension to differentiate it from secondary intracranial hypertension caused by malignancy, IIH should not be considered a benign disorder as symptoms include disabling headaches, obesity, papilledema, and blurred vision, which evolve slowly over time and can cause severe and permanent vision loss in 10% of affected individuals due to pressure on the optic nerve [3,4].

The incidence of IIH in the general population is thought to be about 1–2 per 100,000. Although it does occur in men, the disorder is most common in obese women of childbearing age with an incidence of 10 per 100,000 [1].

Imaging of the brain with MRI is essential in patients with suspected IIH to exclude elevated CSF pressure due to other causes. MRI may show slit-like ventricles or a flattened pituitary suggesting a diffuse accumulation of water in the skull causing optic nerve tortuosity, optic nerve sheath distension [5], and increased CSF clearance activity seen as hypertrophic Paccioni’s granulations which can produce stenosis when located close to the transverse sinus, causing blood retention and a further increase in ICP.

Hyperintensity and increased optic nerve diameter, suggestive of optic nerve oedema, observed in T2-weighted and angio-venous MRI showing uni- or bilateral transverse sinus stenosis due to hypertrophic Pacchioni’s granulations support the diagnosis of IIH [6].

Genetic predisposition to IIH has been previously proposed [7] and indeed several cases of multiple incidences in families have been described [8,9] showing that IIH occurrence within a family is more common than previously believed, and its incidence in families is more common than in the general population. Nevertheless, there does not appear to be a Mendelian pattern of inheritance. IIH would be more likely caused by the concurrence of environmental factors such as overweight in individuals with increased genetic susceptibility [10].

Aquaporins (AQPs) are a family of water-channel proteins, which in many cells provide the main route for bidirectional water movement across the membrane by increasing plasma membrane osmotic permeability [11]. AQPs have been found in different cerebral structures. Among these, the most common are AQP1 and AQP4. The roles of AQPs have been demonstrated in several diseases such as cerebral edema, various central nervous system (CNS) tumours, Alzheimer’s disease, and epilepsy [12], but there are few previous studies on the role of AQPs in IIH and the results are inconclusive.

Here, we study the association between different polymorphisms in AQP1 and AQP4 in order to better understand the role of AQPs in the pathogenesis of and predisposition to IIH.

## 2 Materials and methods

### 2.1 Patients’ samples

A total of 72 Caucasian patients, 55 female (73.6%) and 17 male (23.6%), with a mean age of 39.01 years and an age range of 11–72 years, diagnosed of having IIH according to Friedman criteria [2], were included in the study. All patients had undergone brain MRI, computed tomography scan of the brain, or both. Brain angio-MRI was performed in patients with neuroimaging signs suggestive of transverse sinus venous stenosis in order to ensure that they met the entry criteria of the study. Clinical data included the most representative symptoms of IIH such as headache, papilledema, obesity, sinus stenosis, and intracranial pressure of above 20 mm Hg.

Intracranial pressure was determined by lumbar puncture, in the L4–L5 interspace, with the patient in the supine decubitus position.

The presence of disorders such as metabolic diseases, liver dysfunction, or infectious diseases, as well as other neurological diseases such as tumours or hydrocephalus seen on neuroimaging were considered exclusion criteria. No pregnant women were included.

The results were compared with those of a control group of 94 healthy Caucasian individuals of Spanish origin.

About 5–10 mL of venous blood was obtained from all participants. From these samples, genomic DNA was isolated using an automated nucleic acid purification system (Roche^®^ Nucleic Acid Purification System MagNA Pure Compact).

### 2.2 Genotyping

Five bi-allelic SNPs rs1049305 and rs10244884 in AQ1 and rs2075575, rs3763043, and rs3763040 in AQ4 were selected and genotyped to determine their allele and genotypic frequencies in the study population and controls.

Samples were genotyped using KASP (Kompetitive allele-specific PCR) genotyping technology (Biosearch Technologies^®^) that allows biallelic discrimination of known SNPs and indels, following the manufacturer’s instructions.

### 2.3 Statistical analysis

The results are presented as genotype and allele frequencies for both IIH patients and control groups. The differences in genotype and allele frequency between the two groups were assessed by Pearson’s *χ* ^2^ tests for 2 × 2 (alleles) or 3 × 2 (genotypes) contingency tables. A *P* value < 0.05 was considered statistically significant. We applied Hardy–Weinberg’s law and its equation which states that in an equilibrium population, given allele frequencies *p* and *q*, the genotypic distribution will be *p*2 + 2*pq* + *q*2. In our study, the distribution of each genotype in our population was considered to be in equilibrium when the comparison between the observed and expected genotypic frequencies applying the Hardy–Weinberg law was <0.05.


**Ethical approval:** This research related to human use complied with all the relevant national regulations, and institutional policies, and is in accordance with the tenets of the Helsinki Declaration, and has been approved by the authors’ institutional review board or equivalent committee. All experiments conformed to the Declaration of Helsinki and were approved by the Ethics Committee of Hospital Clínico Universitario de Valladolid.
**Informed consent:** Informed consent has been obtained from all individuals included in this study.

## 3 Results

### 3.1 Clinical findings

The clinical data of the patients are included in Table 1.

**Table 1:** Relation between intracranial pressure and clinic dates

Clinical datesPatients *n* 72 (%)								
Intracranial pressure	Headache		Papilledema		Obesity		Sinus venous stenosis	
	60 (83%)		42 (58%)		37 (51%)		25 (34%)	
	Yes	No	Yes	No	Yes	No	Yes	No
<30 mm Hg	34	12	23	23	14	17	17	29
>30 mm Hg	26	0	19	7	23	18	8	18

Headache was the most frequent symptom, affecting 60 patients (83%). Papilledema was found in 42 patients (58%). Obesity was present in 27 patients (51%). Radiologically, venous sinus stenosis was observed in 25 patients (34%).

Lumbar CSF pressure was elevated in all patients with a range of 20–57 mm Hg and a median of 28.64 mm Hg.

No statistically significant difference was observed between clinical data and intracranial pressure. However, when we compare the data with intracranial pressure equal to or higher than 30 mm Hg, there is a statistically significant difference with headache (*P* = 0.0116). The increase in headaches with increased pressure is a clear cause-and-effect situation. The other clinical data, although related to intracranial pressure, are due to another type of cause.

### 3.2 Analysis of polymorphism

Genotypic and allele frequencies are summarized in Tables 2 and 3, respectively.

**Table 2:** Genotypic frequencies in IIH patients and controls

Polymorphism	Genotypes			*P*
**rs3763040**	**GG**	**GA**	**AA**	
IIH patients	56 (77.8%)	14 (19.4%)	2 (2.8%)	**0.0442**
Controls	56 (59.6%)	32 (34.0%)	6 (6.4%)	
**rs2075575**	**GG**	**GA**	**AA**	
IIH patients	23 (31.94%)	35 (48.61%)	14 (19.44%)	**0.9371**
Controls	28 (29.78%)	46 (48.93%)	20 (21.27%)	
**rs3763043**	**CC**	**CT**	**CC**	
IIH patients	31 (43.05%)	34 (47.22%)	7 (9.72%)	**0.1703**
Controls	50 (53.19%)	31 (32.97%)	13 (13.82%)	
**rs1049305**	**GC**	**GC**	**CC**	
IIH patients	21 (29.16%)	38 (52.77%)	13 (18%)	**0.3296**
Controls	34 (36.17%)	50 (53.19%)	10 (13.51%)	
**rs10244884**	**TT**	**TC**	**CC**	
IIH patients	14 (19.44%)	36 (50.00%)	22 (30.55%)	**0.8437**
Controls	21 (22.34%)	43 (45.74%)	30 (31%)	

A = Adenine; C = Cytosine; G = Guanine; T = Thymine.

**Table 3:** Allele frequencies in IIH patients and controls

Polymorphism	Allele frequencies		*P*
**rs3763040**	**G**	**A**	
IIH patients	126 (87.5%)	18 (12.5%)	**0.0171**
Controls	144 (76.6%)	44 (23.4%)	
**rs2075575**	**G**	**A**	
IIH patients	81 (56.25%)	63 (43.75%)	**0.8065**
Controls	102 (54.25%)	86 (45.74%)	
**rs3763043**	**C**	**T**	
IIH patients	96 (66.66%)	48 (33.33%)	**0.639**
Controls	131 (69.68%)	57 (30.31%)	
**rs1049305**	**G**	**C**	
IIH patients	80 (55.55%)	64 (44.44%)	**0.2253**
Controls	118 (62.76%)	70 (37.23%)	
**rs10244884**	**T**	**C**	
IIH patients	64 (44.44%)	80 (55.55%)	**1**
Controls	85 (45.21%)	103 (54.78%)	

A = Adenine; C = Cytosine; G = Guanine; T = Thymine.

No statistically significant differences were found when comparing allele and genotypic frequencies for SNPs rs2075575 and rs3763043 of AQ4 and rs1049305 and rs10244884 of AQ1. In contrast, differences were observed when comparing the results of the rs3763040 polymorphism of the AQ4 locus. Specifically, differences in genotypic frequencies (*P* = 0.0442) and allele frequencies (*P* = 0.0171) were observed between patients and controls.

**Figure 1:**
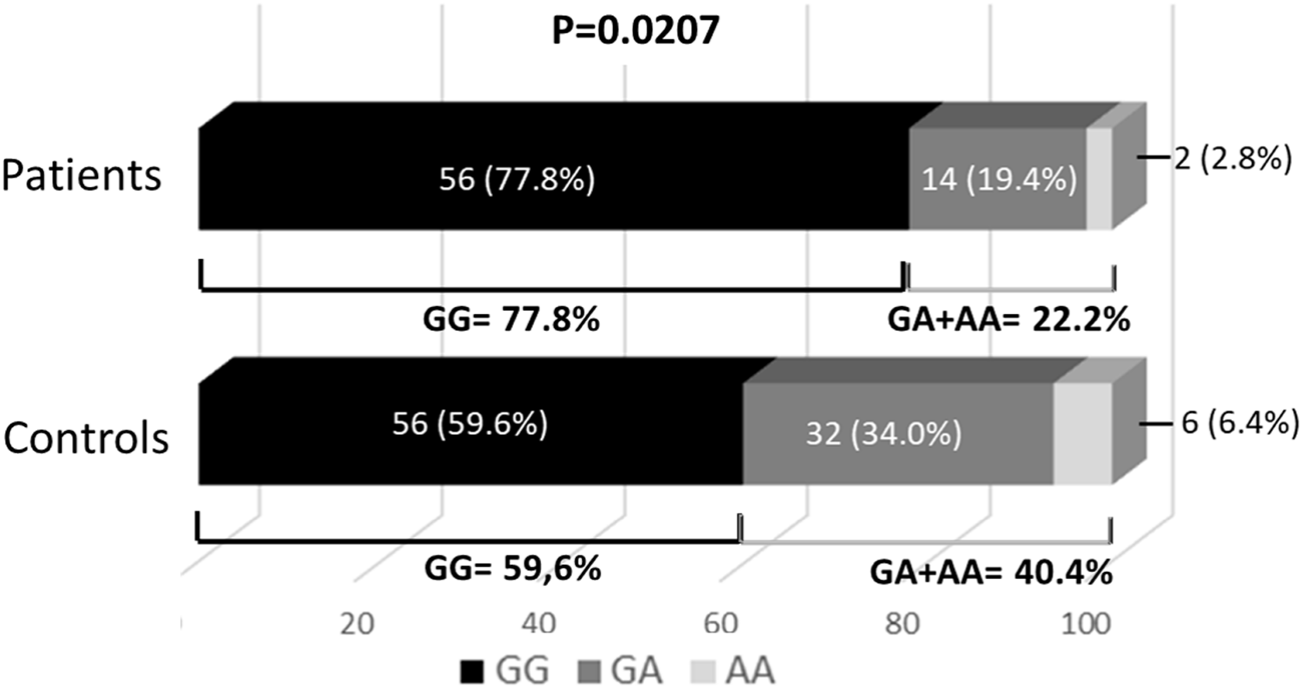
Comparison between individuals carrying the minor allele (A): genotype GA + AA vs homozygotes for the major allele: genotype GG.

Furthermore, when comparing individuals carrying and not carrying the minor allele (AA + GA) individuals vs. GG homozygotes, a statistically significant difference (*P* = 0.0207) was also observed between the groups studied (Figure 1).

The odds ratio of A allele carriers (AA + GA) to GG homozygotes was 2.3750, with a confidence interval of 1.289–4.743 at a 95% confidence level.

No statistically significant differences were found when comparing polymorphism and clinical dates.

## 4 Discussion

The higher incidence of IIH in individuals with family history than in the general population, the reported parent–child transmission, and concordance in siblings [7,13] suggest the existence of genetic susceptibility factors that are inherited in a non-Mendelian multifactorial pattern. IIH would occur when non-genetic triggers act in individuals with increased susceptibility.

AQPs are a family of tetrameric membrane proteins whose primary function is to transport water across cell membranes in response to osmotic gradients created by active solute transport.

Given the known role of AQPs in the regulation of water flow, in the present study, we have analysed whether different genetic polymorphisms of AQPs that are expressed in the brain (namely, AQP1 and AQP4) are related to individual susceptibility to IIH.

We found no association between four of the SNPs studied (the two in AQP1 and two of the three in AQP4) and IIH. However, the study of the rs3763040 SNP in the intron region showed that both allele and genotypic frequencies were different when comparing the patient and control groups. The major allele (G) was more frequent in patients with IIH than in controls, with *P* = 0.0442, so the G allele is associated with an increased risk of developing IIH, especially when found in homozygosis, with patients carrying the minor allele (A) in both homozygosis and heterozygosis showing a lower risk of IIH (*P* = 0.0207) (Figure 1).

AQP4 is the principal AQP in the mammalian brain and is found in supporting cells in the CNS facilitating water movement into and out of the brain [11].

The hypothesis of AQP4 involvement in the pathogenesis and progression of IIH is supported by the observation that AQP4-deficient mice develop greater ICP than wild-type mice when blood–brain barrier disruption is induced [14]. Moreover, acetazolamide is known to inhibit water conduction by AQP-4 [15,16] and its use in IIH patients has a direct effect on papilledema and intracranial pressure, and significantly improves visual field function in patients with IIH [17].

The AQP4 intronic rs3763040 polymorphism has also been related to neuromyelitis optica [18] and to the progression of Alzheimer’s disease [19], meaning that it is an SNP with functional consequences or in linkage disequilibrium with another polymorphism that has functional consequences.

Our case–control genetic study is at odds with another study in 28 Norwegian patients with IIH in which no correlation was found between genetic variants in AQP4 and IIH [20]. In this study, the authors themselves stress that an etiopathogenic link between AQP4 and IIH remains attractive and that further association studies should be performed in larger sample sizes despite the difficulty given the rarity of the condition.

Based on a precise definition of the IIH phenotype and a plausible etiopathogenic hypothesis and its candidate genes, we have conducted a case–control study which, to our knowledge, is the first work to demonstrate an association between genetic variants in AQP4 and IIH defined phenotype, which had an apparent *a priori* hypothesis and a strong biological plausibility.

The association of AQP4 and specifically of its polymorphic variant rs3763040 with IIH should be validated in other ethnic groups to assess more precisely the role of AQP4 in the etiopathogenesis of IIH.

This study is limited by the number of patients (72). Considering that IIH is a rare disease, it is difficult to obtain a larger sample, which would be very useful to validate the results. On the other hand, the findings of statistical significance for the rs3763040 polymorphism of AQP4, without being able to say that it is the cause, open the discussion of its role. Further studies to test the gene, as well as its pathway relationship with other genes, may provide interesting data to determine what appears to be a multifactorial disease.
